# A geochemical view into continental palaeotemperatures of the end-Permian using oxygen and hydrogen isotope composition of secondary silica in chert rubble breccia: Kaibab Formation, Grand Canyon (USA)

**DOI:** 10.1186/s12932-017-0047-y

**Published:** 2018-01-16

**Authors:** Ray Kenny

**Affiliations:** Geosciences Department, Fort Lewis College, Durango, CO 81301 USA

**Keywords:** Oxygen and hydrogen stable isotopes, Chert, Grand Canyon, Authigenic silica, Kaibab Formation, Permian–Triassic Boundary, End Permian, Chert rubble, Breccia, Subaerial exposure surface, Karst

## Abstract

The upper carbonate member of the Kaibab Formation in northern Arizona (USA) was subaerially exposed during the end Permian and contains fractured and zoned chert rubble lag deposits typical of karst topography. The karst chert rubble has secondary (authigenic) silica precipitates suitable for estimating continental weathering temperatures during the end Permian karst event. New oxygen and hydrogen isotope ratios of secondary silica precipitates in the residual rubble breccia: (1) yield continental palaeotemperature estimates between 17 and 22 °C; and, (2) indicate that meteoric water played a role in the crystallization history of the secondary silica. The continental palaeotemperatures presented herein are broadly consistent with a global mean temperature estimate of 18.2 °C for the latest Permian derived from published climate system models. Few data sets are presently available that allow even approximate quantitative estimates of regional continental palaeotemperatures. These data provide a basis for better understanding the end Permian palaeoclimate at a seasonally-tropical latitude along the western shoreline of Pangaea.

## Background

The end Permian subaerial erosion surface exposed at the top of the Kaibab Formation (KF) is important because it occupies a time in Earth history that represents the largest and most severe mass extinction in the Phanerozoic [1–3] resulting in a substantial loss of terrestrial and marine life [4, 5]. A plethora of models and hypotheses have been proposed and discussed [6, 7] to account for the mass extinction event and the catastrophic environmental changes that occurred during the end-Permian event and across the Permian–Triassic transition, including: (1) bolide impacts [8]; (2) catastrophic methane bursts [5, 9–12]; (3) oceanic degassing of hydrogen sulfide [13]; and, (4) massive CO_2_ release from the eruption of Siberian flood basalts [14, 15]. Kidder and Worsley [16] have suggested that a significant increase in atmospheric CO_2_ was present across the Permian–Triassic Boundary (PTB) resulting in global, tundra-free, “warmhouse” climate conditions [17, 18]. Numerous studies have focused on geochemical evidence preserved as biomarkers that point to global euxinic or anoxic marine conditions [19–22]. Chronologic constraints for the end-Permian extinction event and the PTB range from 251.4 to > 254 Ma [23, 24]. Shen et al. [25] suggest that the mass extinction event lasted for ~ 200,000 years with the peak of extinction occurring just before 252.28 ± 0.08 million years ago. Kiehl and Shields [26] used reconstructed palaeogeographical data to produce a comprehensive climate model which showed that the Earth was warmer than the present, which agrees with other palaeodata, climate models, and palaeoenvironmental studies of the end Permian [18, 19, 27–30].

**Fig. 1:**
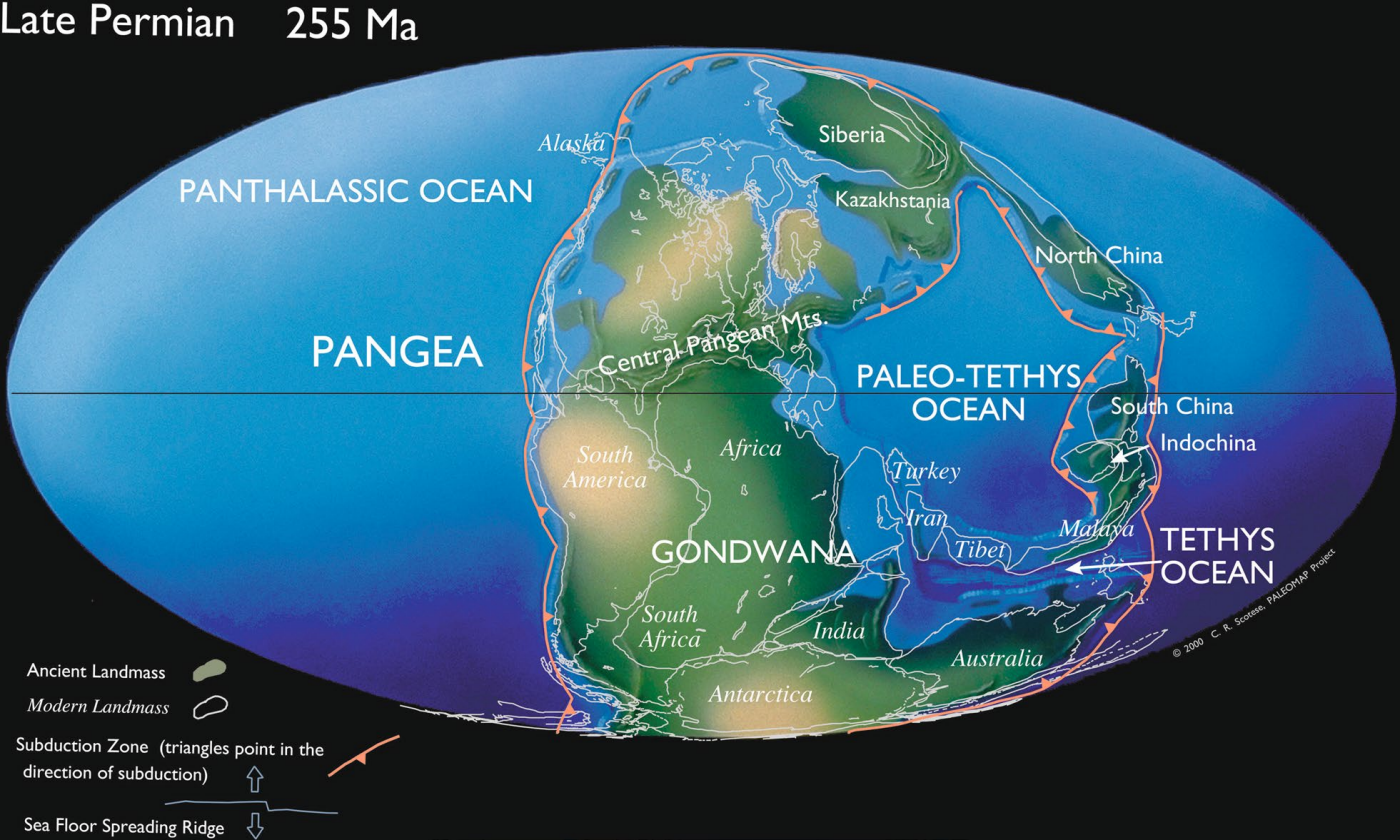
Palaeogeographic map of the Late Permian (255 Ma) (Used with permission [89])

The Permian KF is composed of a variety of lithologic types but predominantly is a shallow marine carbonate with subordinate siliciclastic sediment, chert and gypsum [31–35]. In northern Arizona, the KF is uncomformably overlain by fluvial, tidal flat and non-marine sediment of the lower Triassic Moenkopi Formation. The lower member of the Moenkopi Formation is largely composed of strata formed during and after the first eastward transgression of the Panthalassic sea (Fig. 1), and typically consists of a laterally variable yellowish siltstone, sandstone, and grey chert pebble conglomerate and breccia [36]. The uppermost beds of the KF contain spatially extensive palaeokarst depressions [31] and form a laterally uniform, thick, chert-rubble erosion surface in well-exposed outcrops across much of northern Arizona and the Grand Canyon region. McFadden [37] and McFadden and Knauth [38] have shown that the uppermost rubble breccia of the KF contains highly fractured, zoned, slumped and silicified chert, and they argued that: (1) the rubble breccia resulted from extensive dissolution of the carbonate host rock; (2) long-term subaerial exposure began after eustatic sea level regression of the end Permian event; and, (3) the subaerial karst event spans the PTB and terminated in the lower Triassic Period based on the presence of chert-pebble and chert-rubble conglomerates in lower Triassic fluvial channels, first noted by Huntoon et al. [39].

Most palaeoenvironmental studies of the end-Permian event and the PTB have focused on well-preserved biomarkers in the marine record [40]. In contrast to the large data sets for coastal and oceanic palaeotemperatures, there are few data sets that allow even approximate quantitative estimates of continental (non-marine) palaeotemperatures because well-preserved, terrestrial sedimentary deposits are scarce. Empirical continental evidence predominantly comes from peatlands, palaeosols, and palaeosol carbonate [41–43 and references therein]. However, Peters et al. [44] argue that palaeotemperature estimates of pedogenic carbonates derived from isotopic values have a seasonal variability bias that yield temperature uncertainties. Oxygen and hydrogen isotopic compositions of silica have been successfully used to better understand the crystallization history of chert [45] based on the premise that once the granular, microcrystalline quartz has crystallized the isotopic composition is preserved. Kenny and Knauth [46] and Kenny [47] demonstrated that oxygen and hydrogen isotopic composition of secondary (authigenic) silica crystallized during subaerial exposure surfaces could be used to approximate near-surface continental weathering temperatures. Both crystallization temperatures and the role of meteoric waters in the initial crystallization of silica can be gleaned from the stable isotope values. The research hypothesis of this study is that: (1) secondary, drusy, botryoidal, vug-fill silica precipitated in the chert rubble of the upper KF could be used to estimate approximate, continental quartz crystallization temperatures for the end-Permian event and across the PTB in northern AZ; and, (2) the isotopic composition of the silica could be used to verify that silica in the chert rubble breccia crystallized in the presence of meteoric (non-marine) waters.

**Fig. 2:**
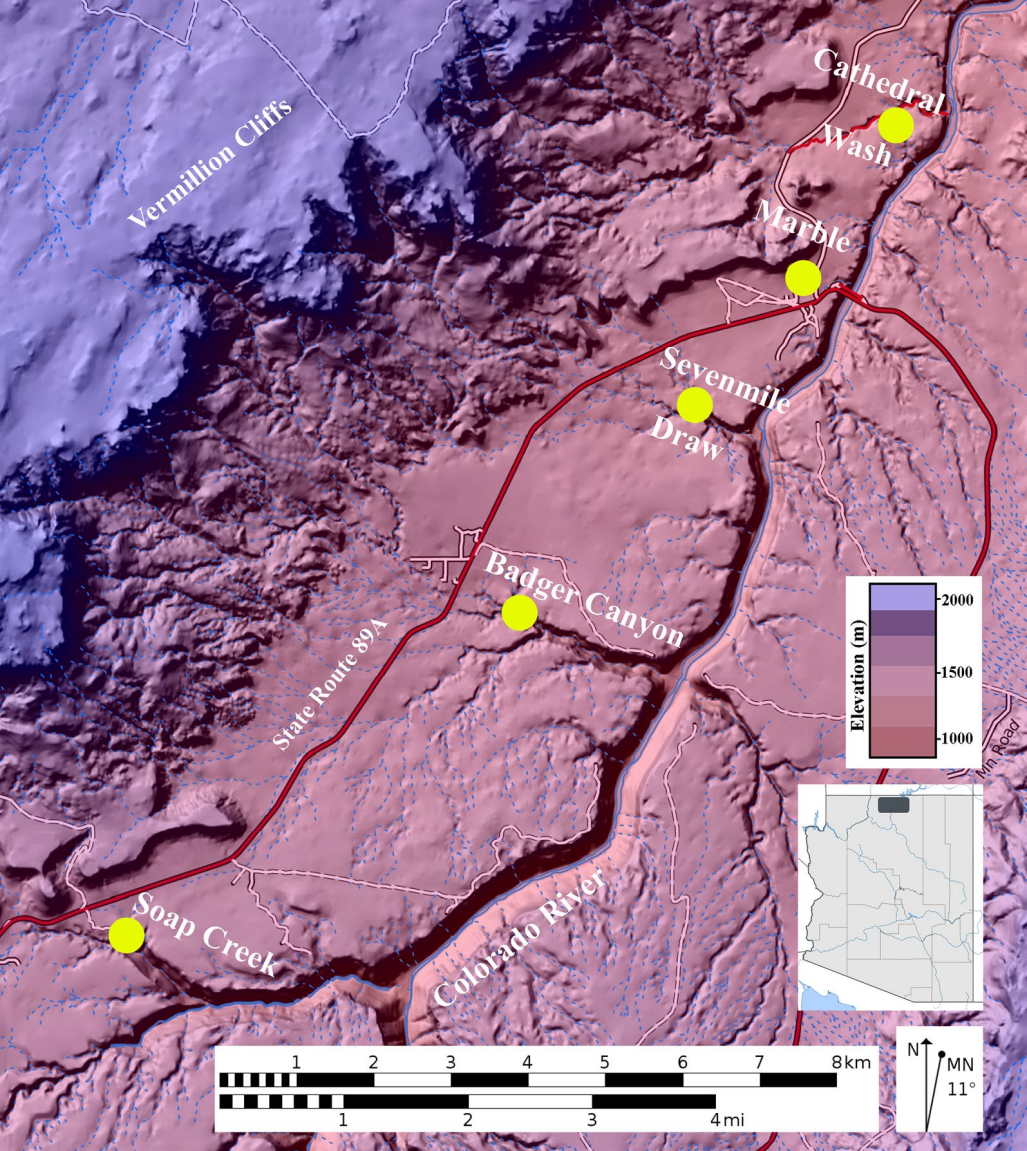
Secondary silica sample locations in the Marble Canyon area of northern Arizona. Samples were extracted from five Kaibab Formation rubble breccia slot canyon sites

**Fig. 3:**
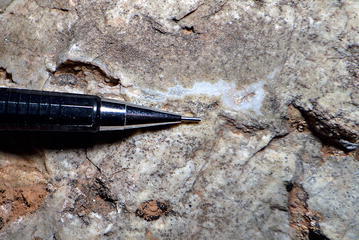
Example of translucent to white secondary silica from the Kaibab Formation chert rubble breccia (Sevenmile Draw). Vugs are typically rimmed with fibrous silica which transition to drusy quartz toward the middle of vugs, interstices or cavities. Mechanical pencil tip (width) is 0.05 mm

**Fig. 4:**
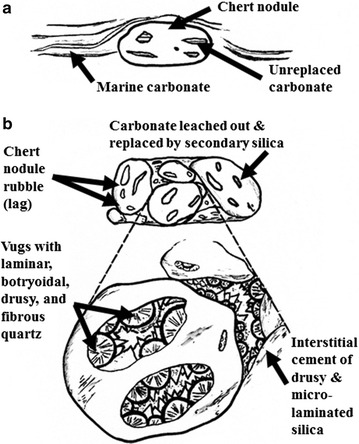
Diagrammatic sketch contrasting chert-rich, marine carbonate rock and secondary silica and chert rubble deposits in a continental karst terrain. **a** Early diagenetic, marine carbonate-draped chert nodule with variable percentage of unreplaced carbonate. **b** Lag accumulation of resistant, slumped and randomly oriented chert nodules in a continental karst terrain. Unreplaced carbonate is leached out and replaced by secondary, cavity-fill silica which consists of fibrous, botryoidal and drusy quartz (Diagram modified from [46])

Silica samples were collected from within chert rubble horizons exposed in narrow and slot canyons draining the southeastern edge of the Vermillion Cliffs near Marble Canyon, AZ (Fig. 2). Samples from several well-exposed outcrop localities contained an abundance of secondary silica phases that were recognizable in hand sample (Fig. 3). Secondary silica samples were reduced to millimeter-size (~ 2–4 mm) silica chips. The silica chips along with a few hyaline microcrystalline quartz crystals, were meticulously extracted from vugs, voids, and lag deposit interstices (Fig. 4). The millimeter-size sample chips were visually inspected, sorted by translucence and color, and analyzed under a binocular microscope for the presence of iron-oxides or other impurities. A hand-held magnet was passed over the sample chips to detect (magnetic) iron-oxide. Samples determined to be free of impurities were selected for stable isotope analysis. Oxygen and hydrogen isotope analyses were conducted following the well-established, in situ laser extraction method of Sharp [48] and Sharp et al. [49] in the University of Texas—Austin stable isotope lab. One silica sample had been previously analyzed in the stable isotope lab at Arizona State University.

## Results

**Fig. 5:**
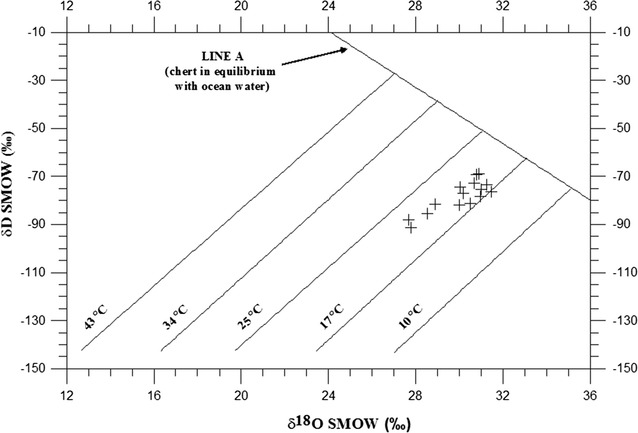
δ^18^O vs δD diagram showing the isotopic compositions of 15 secondary silica samples extracted from Kaibab Formation rubble breccia lag deposits. The oxygen and hydrogen isotope ratio data of secondary silica separates form domains elongated away from Line A. Line A (actually more of a band) is the inferred locus of isotopic compositions of cherts in equilibrium with modern sea water at various temperatures [45]. Silica data elongated away from Line A indicate that meteoric (fresh) waters were involved in the crystallization history of the silica. Silica data are interpreted in terms of palaeotemperatures by comparing them with temperature lines drawn approximately parallel to the meteoric water line [45]. Palaeotemperature estimates for the secondary silica separates range from ~ 17 to 22 °C. The most ^18^O- and D-depleted samples are likely those with the purest amount of authigenic silica. These are represented on the diagram by four samples which plot farthest from Line A and closer to the warmest palaeotemperture estimates of ~ 21–22 °C

Oxygen and hydrogen isotope ratio data of 15 secondary (authigenic) silica separates from the KF are shown in Fig. 5; the data are given in Table 1. A total of five rubble breccia localities were examined and sampled (Fig. 2). All data are reported relative to V-SMOW in standard δ-notation. δ^18^O values represent total (structural) oxygen of chert and silica; δD values are derived from non-surface hydroxyl groups of chert and silica. All analyses have a precision of ± 0.2 and ± 2‰ for δ^18^O and δD, respectively. The present data do not allow for precise error estimates, and the ability to assign temperatures on the order of plus or minus several °C does not presently exist.

**Table 1:** Description, data and location of fifteen Kaibab Formation secondary (authigenic) silica samples extracted from the end-Permian chert rubble breccia (northern Arizona)

δ^18^O SMOW (‰)^a^	δD SMOW (‰)^a^	Description and location of secondary silica samples
30.8	− 74.5	Drusy, vug-fill silica (Marble Canyon; KNB1)
30.6	− 70.0	Drusy, vug-fill silica (Soap Creek; SC2)
29.7	− 87.8	Translucent, microcystalline silica vug-fill (Badger Canyon; BC1)
30.8	− 77.6	Translucent drusy, vug-fill quartz (Marble Canyon; MC22)
29.9	− 76.1	White, translucent granular microcrystalline silica (Badger Canyon; KF1)
30.6	− 73.6	Translucent granular microcrystalline silica (Marble Canyon; K6)
30.99	− 78.0	White, translucent granular microcrystalline silica (Badger Canyon; KF2)
31.01	− 75.7	Translucent, dull botryoidal silica (Sevenmile Draw; K7)
28.52	− 86.2	Translucent, granular microcrystalline silica (Sevenmile Draw; K5)
31.5	− 77.0	White, translucent granular microcrystalline silica (Marble Canyon; NB1)
30.9	− 68.9	Translucent, granular microcrystalline silica (Soap Creek Canyon; SC3)
30.46	− 80.8	Translucent, dull botryoidal silica (Sevenmile Draw; 744)
28.9	− 82.0	White, translucent granular microcrystalline silica (Marble Canyon; NBK)
27.7	− 92.0	White, pale blue fibrous silica (Cathedral Wash; NB3)
27.8	− 87.42	White, translucent granular microcrystalline silica (Cathedral Wash; NB5)

^a^Reproducibility for δ^18^O is ± 0.2‰ and δD is ± 2‰. Reproducibility is based on duplicate runs and standards

The oxygen and hydrogen isotope ratio data derived from KF secondary silica separates form domains elongated away from Line A on a δD-δ^18^O diagram (Fig. 5). Line A is the inferred locus of isotopic compositions of cherts in equilibrium with modern sea water at various temperatures [45]. Silica data are interpreted in terms of palaeotemperatures by comparing them with temperature lines drawn approximately parallel to the meteoric water line as established by Knauth and Epstein [45]. Silica data elongated away from Line A indicate that meteoric (fresh) waters were involved in the crystallization history of the chert and silica. Palaeotemperature estimates for the secondary silica separates range from about 17–22 °C. The most ^18^O- and D-depleted samples are likely those with the purest amount of authigenic silica. These are represented on the diagram by four samples which plot closer to the warmest palaeotemperature estimates of ~ 21–22 °C (Fig. 5). The silica data indicate that the palaeoclimate at the time of silica crystallization was warm.

## Discussion

During subaerial exposure and the development of karst terrain in chert-rich carbonates, insoluble chert nodules accumulate as chert rubble lag deposits [46]. The precipitation of secondary silica in karst terrains occurs when downward-percolating meteoric waters reach the saturation level of quartz (~ 6 ppm) [50] or amorphous silica (~ 80 ppm) while passing through the chert rubble breccia [46, 51]. The dissolution of quartz in water results from a straightforward hydration process to form silicic acid, but the dissolution rate is considerably variable depending upon local environmental conditions [52]. Complexing of silicic acid with certain organic acids, which typically occur in tropical karst environments, has been shown to increase quartz solubility [53]. Livingstone [54] documented that in high-rainfall areas, conditions typically associated with tropical karst environments, significantly higher silica content occurs in waters draining known silica sources. White et al. [55] suggested that during karst development, quartz first hydrated to the more soluble opal mineral. However, Martini [56] and Chalcraft and Pye [57] showed that the transformation of quartz to opal is not thermodynamically possible at surface conditions. MacKenzie and Gees [58] grew 10 μm-sized euhedral microquartz at 20 °C in 2 years, which suggest that there is no a priori reason that quartz cannot form from silica solutions carrying > 4 ppm dissolved silica [58]. Rimstidt [59] predicted from experiments done in *pure water* that the solubility of quartz would be 11 ppm ± 1.1 ppm at 25 °C. Knauth [60] argued that there is no compelling evidence that opal is a necessary precursor to microquartz precipitates in any epicontinental replacement cherts. The prominence of drusy and botryoidal silica phases in KF chert rubble vugs suggest that quartz precipitated directly without opaline percursors, and stable isotopic data show clearly that the microquartz sampled from the paleokarst horizons precipitated from waters that had a meteoric water component. The source of the silica can come from dissolution of fine-grained quartz leached out of carbonate during infiltration and/or capillary rise of meteoric water. Skotnicki and Knauth [61] documented nearly complete replacement of karst features and flowstones by secondary silica phases in the Mescal Limestone of northern Arizona. Those authors suggested that the widespread replacement resulted from intense weathering of the Mescal carbonate and the presence/release of an abundant silica source. On a smaller scale, Hill and Forti [62] suggested that calcite replaced by silica in some speleothems likely resulted from pH fluctuation. Although the precise mechanism for precipitation of secondary silica under subaerial erosion surfaces is not entirely clear, stable isotopic analysis of silica unequivocally indicate that the silica did not precipitate in the presence of marine water and that meteoric water played a role in the crystallization of secondary silica.

Analyses of oxygen and hydrogen isotope ratios of chert and silica have yielded reasonable and reproducible palaeoclimate estimates that have been independently verified with proxy data which are resistant to alteration and isotopic exchange, including clay minerals and iron-oxyhydroxide material [63]. Oxygen and hydrogen isotope ratios of chert and silica reliably record the isotopic composition of total oxygen and trace hydroxyl groups preserved in chert at the time of crystallization [45]. Approximate palaeoclimate estimates based on analyses of oxygen and hydrogen isotope ratios of marine chert are well established [45, 64, 65]. Kenny and Knauth [46] demonstrated that oxygen and hydrogen isotopic composition of secondary (authigenic) silica precipitated in palaeokarst chert lags could be used to estimate near-surface continental weathering temperatures as far back as the Late Proterozoic. Kenny [47] reported continental weathering temperatures inferred from oxygen and hydrogen measurements of secondary silica precipitated during a tropical karst event that developed on the Mississippian Redwall Limestone of northern AZ (USA). Abruzzese et al. [63] suggested that oxygen and hydrogen isotope ratios of freshwater chert could be used as an indicator of regional climatic variation in the Cenozoic and concluded that early diagenetic chert likely record surface conditions. Despite the fact that chert and silica have been successfully used to estimate palaeoclimatic conditions, the temperature assignments made with the Knauth and Epstein [45] approach are subject to several uncertainties. (1) The curve for quartz-water isotope fractionation with temperature has not been experimentally verified for low temperatures. The calculated temperature lines, used in this approach (Fig. 5), are extrapolated from well understood, slightly higher-temperature, quartz-water curves [45]. Sharp et al. [66] investigated Δ^17^O variations in low temperature quartz samples in an effort to constrain the temperature of the water from which the quartz precipitated. The authors outlined a number of assumptions and derived a quartz-water fractionation-temperature relationship for low temperatures. This new quartz-water fractionation-relationship may yield a 1 or 2 °C decrease in temperature estimates relative to the original temperature extrapolations made by Knauth and Epstein [45]. A 1 or 2 °C temperature change is within the error range for the current temperature assignments, which is on the order of plus or minus a few °C. As such, the temperature estimates for this and previous studies are imperceptibly impacted by the new quartz-water fractionation-temperature relationship for low temperature developed by Sharp et al. [66]. If future work on low-temperature, quartz-water fractionation-temperature relationships yield significantly different temperature estimates, then the temperatures derived from our approach will need to be adjusted accordingly. (2) Stable isotope ratios of chert must be preserved through time. Microorganisms embedded in Precambrian chert attest to the chemical integrity and physical stability of chert [64, 67, 68]. Remarkable preservation of microorganisms [e.g. 68–72] and previous oxygen and hydrogen isotope ratio studies of chert by Kenny and Knauth [46] suggest excellent preservation of original isotopic values of chert dating from the Late Proterozoic. Brasier et al. [73] argued that some of the oldest, previously reported bacterial microfossils from the ~ 3.5 Ga Apex Group chert may be geochemical artefacts; other Late Proterozoic microfossils preserved in chert have not been disputed. New research by Schopf et al. [68] has now confirmed the existence of bacteria and microbes in the 3.465 billion year old, Western Australian Apex Group chert. (3) The temperature estimates in Fig. 5 also depend on the assumption that δ^18^O values of sea water have not changed significantly throughout geologic time. The generally lower δ^18^O values of ancient carbonates (e.g., from the Silurian Period) have been used as an argument that δ^18^O values of the past oceans were lower than modern values [74, 75]. Knauth and Roberts [76] provide arguments that they consider fatal to using carbonates to monitor the oxygen isotope composition of past seawater. Knauth and Roberts [76] presented data, including direct analysis of unaltered ocean water preserved in halite, which precludes the proposed ~ 5–6‰ oxygen isotope ratio changes in seawater as far back as the Silurian Period. In order to adequately determine the diagenetic history of carbonate samples used to monitor the oxygen isotopic composition of past seawater, both ^13^C and ^18^O co-variant values are needed. Veizer and Prokoph [75] analyzed oxygen isotope ratios of carbonates to propose secular oxygen isotope ratio changes in ocean water during the Phanerozoic, but co-variant ^13^C values for the oxygen isotope measurements have not been included in their published data set. Both ^13^C and ^18^O co-variant values are needed to determine if the platform carbonates are original precipitates, have been diagenetically altered at the molecular level, or have been partially altered by meteoric waters during the transformation of the host sediment into limestone [e.g., 77]. Zempolich et al. [78] and Kenny and Knauth [79] analyzed co-variant carbon and oxygen isotope ratios of Proterozoic Beck Spring carbonates to argue that the Late Proterozoic (Beck Spring) ocean was not significantly different from modern sea water. Clumped isotope thermometery is a new approach that uses isotopologues (which are independent of the bulk isotopic composition) to examine the temperature dependence of bond formation between two rare, heavy isotopes within a single molecule to independently determine the δ^18^O value of the fluid in a carbonate sample [80–83]. Cummins et al. [84] used clumped isotope analysis to address the complicated uncertainties related to diagenetic alteration of δ^18^O in carbonates used to estimate the oxygen isotopic composition of past seawater. Cummins et al. [84] measured a large suite of well-preserved Silurian (ca. 433 Ma) carbonate fossils and determined that Silurian oceans had oxygen isotopic composition similar to the modern ocean. Clumped isotope research of non-icehouse carbonates from other geologic time periods has also been reported yielding similar results [85]. Collectively, the clumped isotope research largely supports previous studies by Knauth and Epstein [45] and Knauth and Roberts [76] which suggest that the δ^18^O of Earth’s ocean waters, during non-icehouse conditions, have remained broadly consistent through the Late Proterozoic. As such, palaeotemperature estimates based on oxygen and hydrogen isotope ratios of chert and silica remain valid until a compelling argument can be presented to the contrary.

Other environmental conditions can also impact the oxygen isotope values of chert and silica. (1) Enrichment of ^18^O in chert may result if silica precipitation occurred under evaporative conditions. Abruzzese et al. [63] documented a large range of oxygen isotope values (~ 20‰) from Eocene and Miocene Epoch lacustrine and fresh water chert which they attributed to large-scale changes in the isotopic composition of lake water due to evaporation. The relatively narrow range of oxygen values of secondary silica from the KF (< 5‰) suggest that evaporative processes were likely insignificant and evaporative enrichment was likely minimal during the crystallization history of the chert. (2) Elevated temperatures from post-precipitation metamorphic processes could produce low oxygen and hydrogen isotope ratios of chert and silica. Metamorphic processes capable of altering oxygen and hydrogen values result in notable and visible changes to silica, including: (a) recrystallization of silica and the formation of mega quartz (> 35 μm); and, (b) thermal annealing of macro- and micro-morphological silica fabrics. Field samples of silica from the KF chert rubble horizons retained drusy, botryoidal and vug-fill silica phases; no pervasive silica-filled veins or post-precipitation alteration was observed. Horizontally stratified sedimentary layers of the KF not influenced by karst processes show no signs of metamorphism and no major faults transect the sample locations. Moreover, the observed isotope values are significantly cooler than if chert had been pervasively altered by hydrothermal fluids which have been measured as low as (~ 100 °C) but which are often substantially hotter [86]. The location and geology of the study area, coupled with the well-preserved silica forms, indicate that the area has not been altered by hydrothermal activity or post-precipitation metamorphic processes, and alteration of original stable isotope values is unlikely.

The KF of northern Arizona was located several degrees north of the palaeoequator along the western shoreline of Pangaea (Fig. 1) during PTB [87–89] which placed the KF in a seasonal, tropical ocean setting [30]. Equatorial winds impacted the western shores of Pangaea across the Panthalassic Ocean and likely would have produced elevated annual precipitation amounts in excess of 2000 mm per year during the latest Permian [30]. Elevated precipitation in a seasonal tropical ocean setting would have enhanced terrestrial weathering, released a substantial volume of silica, and accelerated karst processes producing the aerially extensive chert rubble breccia of the KF. Roscher et al. [30] ran climate models on the latest Permian and determined that the minimum global annual mean temperature for the *pre*-mass extinction event was ~ 18.2 °C (~ 3 °C above the current average). Kiehl and Shields [26] modeled terrestrial seasonal mean surface air temperatures for the late Permian *during* the climate perturbation (mass extinction) event, and estimated a global seasonal mean average temperature ~ 7.98 °C *above* the current average (~ 23.2 °C). Coastal regions of western Pangaea were influenced by maritime climate conditions that likely buffered temperature extremes during the end Permian. Results from this study indicate that secondary silica along the western shoreline of Pangaea, at the palaeolatitude of the KF, crystallized at temperatures between ~ 17 and 22 °C. The palaeotemperature estimates from this study broadly agree with the global mean temperature estimates modeled by Roscher et al. [30], as well as the seasonal mean surface air temperatures during the climate perturbation (mass extinction) event modeled by Kiehl and Shields [26].

## Summary and conclusions

Drusy, botryoidal and vug-fill silica from the uppermost members of the Kaibab Formation chert rubble in the Marble Canyon (AZ) area contain silica suitable for palaeoclimate analysis inferred from oxygen and hydrogen isotope measurements. Application of the Knauth and Epstein [45] method for assigning palaeotemperatures to isotopic values of chert and silica yield a long-term palaeotemperature range of ~ 17–22 °C during the end Permian a few degrees north of the palaeoequator along the western shoreline of Pangaea. Accuracy of these palaeotemperature estimates is subject to certain assumptions inherent in the interpretation of isotopic data. The oxygen and hydrogen isotope values clearly indicate that meteoric (non-marine) waters were present during secondary silica crystallization. Oxygen and hydrogen isotope values of secondary silica from the Kaibab Formation chert rubble breccia are broadly consistent with palaeogeographic climate models that estimate an ~ 18.2 °C minimum global annual mean temperature for the latest Permian.

## Abbreviations

KF: Kaibab Formation
PTB: Permian–Triassic Boundary
AZ: Arizona
